# Reducing Rat Lungworm Disease in Hawai'i Through a Collaborative Partnership With K-12 School Garden and Agriculture Projects

**DOI:** 10.3389/fpubh.2018.00203

**Published:** 2018-07-24

**Authors:** Kathleen Howe, Jenny Bach, Myles DeCoito, Shari Frias, Rebecca Hatch, Susan Jarvi

**Affiliations:** ^1^Department of Pharmaceutical Sciences, Daniel K. Inouye College of Pharmacy, University of Hawai'i, Hilo, HI, United States; ^2^Hawai'i Department of Education, Laupahoehoe Public Charter School, Laupahoehoe, HI, United States; ^3^Hawai'i Department of Education, Ka'Umeke Ka'eo Public Charter School, Hilo, HI, United States; ^4^Hawai'i Department of Education, The Volcano School of Arts and Sciences, Volcano, HI, United States

**Keywords:** rat lungworm disease, *Angiostrongylus cantonensis*, Hawai'i, school gardens, integrated pest management

## Abstract

A recent increase in serious cases of rat lungworm disease impacts Hawai'i's agriculture and human health. Slugs and snails, agricultural pests, are intermediate hosts of *Angiostrongylus cantonensis* the rat lungworm. Infection by this parasitic nematode is the leading cause of eosinophilic meningitis globally. Infection can result from ingestion of infected produce and has caused chronic neurological problems, disability, coma, and death. There are over 200 K-12 school and youth garden, and agriculture projects throughout the Hawaiian Islands. This existing network provides an avenue for teacher and student involvement in community health education and host control programs. We collaborated with five Hawai'i Island schools connected with the Hawai'i Island School Garden Network to develop an integrated pest management plan for the control of invasive, intermediate hosts of *A. cantonensis*. Curricula relating to rat lungworm for grades 5–8 that support student academic achievement with a focus on science, technology, engineering, art, and math were developed. The management plan trialed the use of five different materials for shelters, which provided refuge for and easy removal of unwanted slugs and snails. Over 4,000 invasive slugs and snails were removed. Students learned how to safely dispatch pests and they collected data on species found, numbers of species removed, and shelter-type capture rates. Using the arts, students shared information at school and within their family and community. A written management plan, eleven lesson plans, and auxiliary materials are now available online. A concerted effort is needed to reduce parasite hosts if we are to reduce human cases of disease and restore public faith in local agriculture. Use of the established school garden network is an ideal avenue through which to educate the public and develop solutions for this public health problem.

## Introduction

Hawai'i may seem a tropical agricultural breadbasket, however, due to its extreme isolation, food security is an important concern. Concerted efforts on the part of the state have made good progress in addressing the issue but have been stymied by agriculture-related, disease-causing organisms. The executive summary of the *Increased Food Security and Food Self-Sufficiency Strategy*^1^ prepared in 2012 by Hawai'i state agencies states an estimated 85–90% of Hawai'i's food is imported. This document sets actions, policies, and objectives to increase production, demand, and access to locally grown foods, and provides policy and organizational support to meet food self-sufficiency needs. The strategy has generated statewide support for local farmers and inspired the Grow Local/Eat Local movement with a noticed increase in farmers' markets and school and youth garden projects, and recently Hawai'i approved the Hawai'i Farm to School Act, which supplies the school meal program with foods procured from local farms. The health benefits of consuming fresh, locally-grown produce cannot be denied. At the same time, however, a vertebrate parasitic nematode carried by slugs and snails that can cause human infection and disease has created a food safety challenge in Hawai'i. Angiostrongyliasis, or rat lungworm disease, caused by *Angiostrongylus cantonensis* the rat lungworm, is established in 30 countries including the United States. In addition to Hawai'i, it has been found in Florida, Louisiana, Texas, and Oklahoma, and its range is expected to expand with climate change (1–4). Angiotstrongyliasis is the leading cause of eosinophilic meningitis globally (1).

The lifecycle of this parasitic nematode begins and ends in the definitive host, the rat. Larvae undergo five developmental stages (L1–L5). Intermediate hosts, slugs and snails, eat rat feces infected with first stage (L1) *A. cantonensis* and harbor early developmental larval stages (L1–L3). It is the third stage (L3) larvae which normally infect the rat, as rats eat slugs and snails, but may also infect accidental hosts (i.e., humans, primates, horses, dogs, some bird species, etc.). In the definitive host, the ingested, microscopic L3 survive the acidic environment of the stomach, burrow through the intestinal wall, enter the blood stream, and migrate to the central nervous system (CNS). There they become macroscopic, growing from fourth (L4) to fifth (L5) stage larvae, after which the young adults leave the brain and finish maturation, entering the heart, pulmonary artery, and lungs where reproduction occurs (1, 2). While the intrusion into the CNS does not seem to affect the rat, when an accidental host is infected neurological damage can result from the parasite itself and the immune system response to the parasite (2). The Center for Disease Control (CDC) states that angiostrongyliasis is usually flu-like and self-resolving, with rare serious cases that can end in disability, coma, or death. In Hawai'i, multiple serious cases are occuring on a yearly basis. Victims are reporting severe, debilitating, and unresolving neurological pain, especially paresthesia. Infections in children have resulted in blindness, developmental delays, and death, and diplopia, paralysis, coma, and death in adults (5–7). Public support of and exports from Hawai'i farmers have been severely impacted. Reports of residents and tourists contracting the disease have resulted in negative press. There has been great concern expressed by residents regarding the disease and lack of information for prevention, diagnosis, treatment, and control.

The first human cases of angiostrongyliasis in Hawai'i were reported in 1959 and 1960 on Oahu (8). A total of 19 cases were reported statewide between 1959 and 1965 (3). From 1966 to 2000 there are no state records of cases (7). The 2007 Hotchberg et al. study (9) investigated cases of eosinophilic meningitis in Hawai'i from 2001 to 2005 and attributed 24 of 83 cases to angiostrongyliasis. In 2007 the HDOH began tracking angiostrongyliasis and two cases were reported. In 2008 cases jumped to eight, and cases have been reported every year since that time with a noticeable increase occuring in 2016 (11 cases) and 2017, when cases increased to 18 laboratory confirmed and three probable cases being reported (7). As a definitive diagnosis requires a spinal tap and visualization of *A. cantonensis* in the spinal fluid or PCR results confirming parasite presence (10), it is agreed that the case numbers are being underreported. The majority of cases reported are from Hawai'i Island, however cases have been reported on all of the major Hawaiian Islands with the exception of Molokai (3).

The consumption of raw or undercooked snails or paratenic hosts is generally the route of angiostrongyliasis transmission in Southeast Asia, but it is usually assumed transmission in Hawai'i is due to the accidental consumption of an infected slug or snail, or piece of slug or snail, on raw, unwashed produce (1, 7). Infection has also been traced to drinking water from a hose or an uncovered beverage contaminated by a slug (7). A mouse study demonstrated that infection can occur across skin, through lacerations, abrasions, or conjunctival tissue (eye, vaginal, anal) (11). This may be a disease transmission pathway in areas where rainwater catchment use is prevalent, such as on Hawai'i Island. There is no federal or state oversight of rainwater catchment in Hawai'i, however many rely on it, especially in areas where angiostrongyliasis cases are being reported. An estimated 30,000–60,000 people rely on rainwater catchment systems for their household water supply, primarily in the Puna, Ka'u, South Kona, and Hamakua Districts on Hawai'i Island [(12); reviewed in: (13)]. No study has been done to determine the efficacy of sediment filters of varying micron size or UV light to block the entry of live *A. cantonensis* into the household water supply. Residents and commercial catchment tank cleaners often report finding slugs and snails crawling on or into tanks and finding drowned slugs/snails in tanks. That drowned slugs and snails can shed live, infective parasites is documented (14–17). A recent survey on Hawai'i Island showed only 66% of catchment systems were expected to produce water that was safe (18).

The Puna District was the entry point for an effective intermediate host, the semi-slug *Parmarion martensi* (19). Any gastropod can potentially serve as an intermediate host, including native snails, however the semi-slug is unusual in behavior, has a high infection rate (65–78%), and carries high parasite loads (19–21). The arrival of this slug around the turn of the millenium correlates with the increase of cases of angiostrongyliasis on Hawai'i Island. Recently angiostrongyliasis cases increased on Maui, leading to the discovery that the semi-slug, formerly thought to only be established on O'ahu and Hawai'i islands, is now established on Maui (3). Infection in rats on Hawai'i Island is also high. Wild rats trapped in the Puna District (*n* = 37) showed 100% infection by visual and molecular analysis (20). A recent study of wild rats (*n* = 545) trapped primarily near the city of Hilo in the South Hilo District, which adjoins the Puna District, reported 93.9% to be positive for RLW infection by either visual and/or molecular inspection (22). Infection rates in definitive and intermediate host organisms in Hawai'i are among the highest worldwide (2, 13).

The conditions described create the perfect conditions for the spread of the disease and a real need for public education and a campaign to encourage the control of hosts of *A. cantonensis*. Education of rural communities of different demographic backgrounds, such as found in Hawai'i, can be challenging (23). Incorporating education into the K-12 school system was first discussed at the 2011 International Rat Lungworm Workshop in Honolulu, Hawai'i. Workshop participants came from six countries including the USA, with personnel from the CDC, the HDOH, the Pacific Basin Agriculture Resource Center (USDA), and the University of Hawai'i Manoa and Hilo in attendance. It was agreed that public education was a priority and one pathway for providing education for hard-to-reach, rural communities was to involve children in the effort by developing STEM (science, technology, engineering, and math) curricula relating to the rat lungworm and rat lungworm disease (24). The Hawai'i Island Rat Lungworm Working Group at the University of Hawai'i, Hilo (UHH) Daniel K. Inouye College of Pharmacy (DKICP) was formed in 2012 with the intention of addressing gaps in rat lungworm disease research and education in Hawai'i. In 2013 the group published “The Mystery of Rat Lungworm Disease,” which provides lifecycle and prevention information. Aligned with 2nd grade science standards and developed in collaboration with teachers from local public schools, over 13,000 copies of this activity book have been distributed statewide. The Hawai'i Island Rat Lungworm Working Group next began to address the need for a control plan for intermediate hosts of *A. cantonensis*, a better understanding of non-native gastropod populations, and advanced lessons relating to the rat lungworm with a focus on STEAM (science, technology, engineering, arts, and math) subjects for higher grade levels.

## Materials and methods

**Figure 1:**
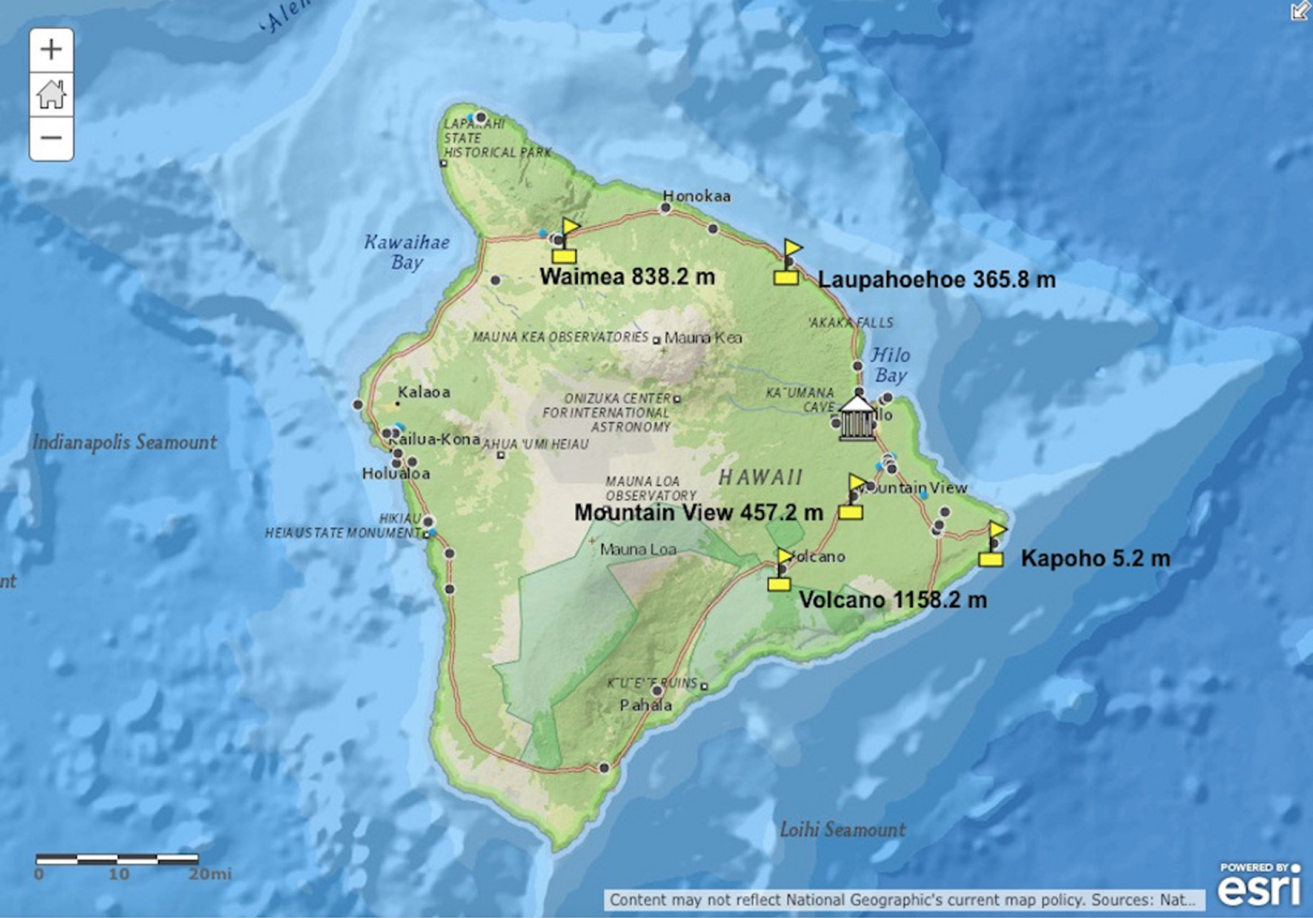
Map of the partner school locations and elevations on Hawai'i Island. Partner schools are shown with yellow flags, the University of Hawai'i at Hilo is depicted with the white building. The black and blue dots show the locations of other public and private K-12 schools on Hawai'i Island.

Five public charter schools located on the windward side of Hawai'i Island with school gardens and members of the Hawai'i Island School Garden Network were chosen to participate in a pilot project to develop an integrated pest management (IPM) plan for the control of non-indigenous slugs and snails and lessons related to the parasite, parasite hosts, and disease transmission, symptoms, treatment, and prevention. The lessons developed were based on peer-reviewed studies, the most recent results of *A. cantonensis* research conducted at UHH, DKICP, and national and international collaborators. The lessons were designed to provide deep learning for students who could become educators for their communities. Schools on the windward side of Hawai'i Island with locations representing different elevations and geographically separate areas were selected to gain a better understanding of non-indigenous species presence and population compositions (Figure 1). The participating schools included two high elevation schools (838.2 m, 1158.2 m), two mid-elevation schools (365.8 m, 457.2 m), and one low-elevation school (5.2 m).

The participating schools serve rural populations from modest to low-income families and ethnically diverse populations, which is representative of much of Hawai'i Island. School garden and classroom teachers and students from grades 5, 6, 7, and 8 participated in the project. Students were asked by an informal show of hands what they knew about rat lungworm disease, if they knew anyone who had had the disease, and if their household water was supplied by rainwater catchment. Students were also questioned as to their knowledge of the parasite, its life cycle, and its hosts prior to lessons given. The project was approved by school principals as it supported school gardens and agriculture projects, protected rural community health, involved place-based learning, and contributed to student academic achievement in STEAM subjects.

**Figure 2:**
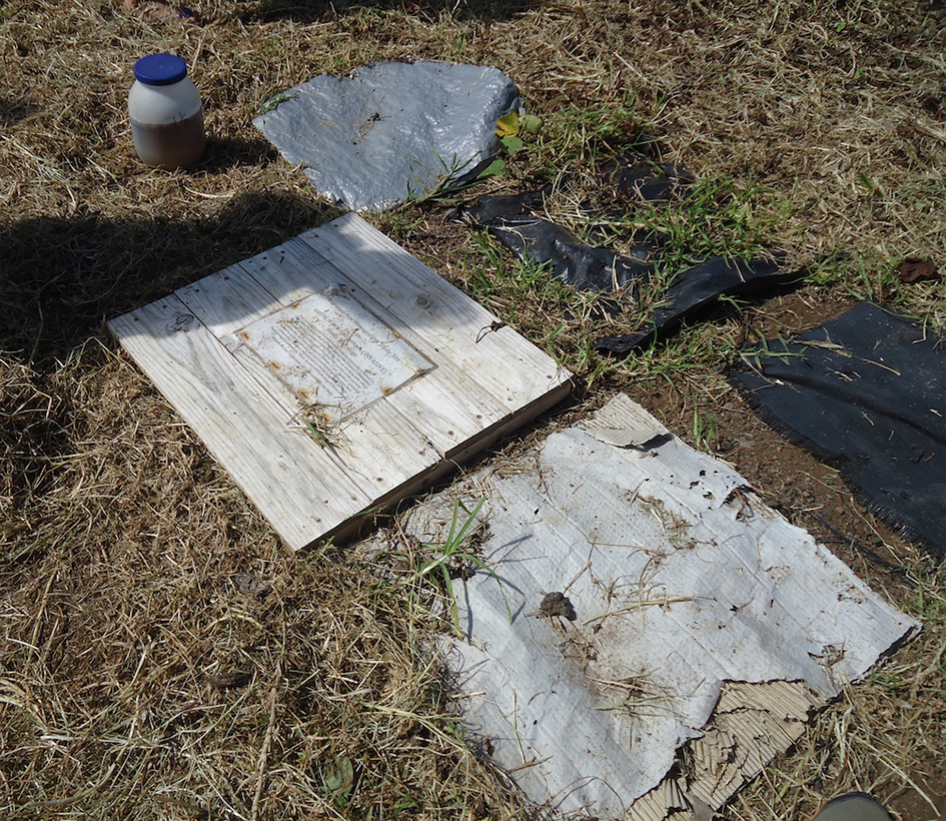
Shelters were 0.61 m^2^ and were made of five different materials (from top moving clockwise in the photo); an insulated reflective tarp, black plastic, weed cloth, cardboard, and a raised wooden board. Two sets of the five shelter-types were placed in each school garden, and the sets were placed at as great a distance from each other as the space afforded. Shelters, excluding the raised wooden board, were fastened to the ground with metal anchor pins.

An IPM plan was trialed and data was collected. IPM uses the least toxic methods to control pests and is therefore desirable. The IPM plan used was modeled after that developed by University of California Statewide IPM Program and included the use of shelters for trapping and hand-picking for control of non-indigenous slugs, snails, and flatworms. Flatworms were included as target pests as they are predacious on slugs and snails and are paratenic hosts of *A. cantonensis* (2). The shelters were 0.61 m^2^ and materials included a raised wooden board, cardboard, black plastic, weed cloth (mesh), and an insulated, reflective tarp. The ground under the shelter was reasonably cleared of vegetation and the soil under the shelter was moistened. Two sets of the five shelter-types were made for each school and the sets were placed in the garden area at maximal distances from each other as the space afforded (Figure 2). Rain gauges were supplied to all partner schools. A master data sheet was created, and copies were given to all participating classes. Identification cards with photos of invasive gastropods common to Hawai'i were given to each school. Schools were visited weekly for the first half of the fall semester, biweekly for the second half of the fall semester, and monthly for the spring semester or as requested by teachers or affected by school/class schedules.

Shelter checks and data collection were done during school visits by students supervised by the project leader. The master data sheet allowed for consistent data recording. Teachers and students were encouraged to check shelters and collect data without project leader support during the second semester. Information collected included shelter-type capture rate, species, species numbers, rainfall amount, date, and name(s) of person(s) recording the information. Species were recorded by using the first three letters of each of the scientific name of the genus and species (i.e., *Parmarion martensis* = PAR MAR) and tic marks were used to record numbers of each species. The project leader aided students with identification of slugs and snails. A comment space on the sheet allowed for recording observations of interest. Safety measures were emphasized. Students wore gloves when participating in activities. A “slug jug” with ingredients to make a 15% saltwater mixture and tongs were given to each school and students were instructed to label the jug and include a safety warning. Organisms captured were dispatched into the saline solution and students were instructed not to look directly into the slug jug when depositing pests to avoid any potential contact with splashed liquid. Target pests were added to the jug until it was deemed ready to empty, readiness based on numbers added, or smell. The contents were left for at least 1 week after the addition of the last organism and the slug jug was emptied onto gravel or rocky surfaces where vegetation was not desired and away from areas of student traffic.

The education format was place-based with standards-aligned STEAM content, integrating school garden and classroom activities. Place-based education is supported and included in Hawai'i's efforts to improve education through the integration of *Every Student Succeeds Act* of 2015^2^ The lesson topics were related to life cycles, gastropod biology, classification and identification, paratenic hosts, native snails, data collection, reporting using ArcGIS online software, and disease transmission, treatment, and prevention. The ArcGIS online story-mapping software is free to all K-12 schools in Hawai'i and can be used to share information within a group, or story-maps can be published for public viewing. Partner schools were encouraged to apply for and download the free software. Participating classes from all schools were expected to develop methods to share the information learned with other classes not involved and with their families. In addition to using the place-based methodology for education, the project espoused student team-work, hands-on activities, citizen science, and a train-the-trainers approach to build a team of adept student researchers/community educators to aid in a statewide effort to rapidly increase our understanding of non-indigenous intermediate host populations and control effectiveness, and public awareness of angiostrongyliasis.

## Results

A total of 85 classroom visits were made during the school year to the five partner schools with 175 students and six teachers participating. Grade levels involved were primarily grades 5–8 but a teacher at one school requested grades 9 (biology), and 12 (environmental science) to be included and the lessons and activities were adapted for these upper grades. A total of 4116 non-native terrestrial slugs, snails, and flatworms were recorded and safely removed from the partner schools from mid-August to mid-December 2015. Over 3,100 were removed from one campus, which had an infestation of *Cornu aspersum*, the European garden snail. It should be noted that the area on Hawai'i Island where the school is located is one of the most important agricultural areas for cool weather crop production for the state of Hawai'i. Whole-campus searches were conducted at this school only due to the intensity of the population, the potential risk to students, and the damage the snails were causing to the native plantings on the campus. Collection of this species in the campus area was conducted by the 12th grade class and these students wrote a slug/snail management plan for their school, while sixth graders controlled in the garden area. The 9th grade biology students focused on the disease presentation, diagnosis, and treatment, and compared angiostrongyliasis with other diseases or injuries causing brain degeneration or injury.

Safety was mandatory, and students involved in removing target species wore gloves. Student teams collected data. Information on species, number, and shelter type were recorded on the data sheet, while team members checked under the shelters (or in vegetation or under or on objects including buildings). Target species were captured using tongs or chopsticks and put into slug jugs. Students became adept at collecting data and identifying the more common non-native species. Species compositions were similar at the two mid-elevation schools with some crossover between low elevation species such as *Achatina fulica*, the giant African snail, and high elevation species such as *Limax maximus*, the leopard slug. The semi-slug, *P. martensi*, which is a tropical invasive from Southeast Asia, was shown to be moving into higher elevations. At the beginning of the project it was absent at the 457.2 m location school but by the end of the first year several specimens of this species were found. The species composition at the Waimea location (838.2 m), where the European garden snail was present, was very different than at other schools. Overall this snail accounted for 75.5% of all organisms removed at all schools followed by *Veronicella cubensis*, the Cuban slug (8.3%), *Derocersas* spp. including the marsh slug and gray garden slug (6.3%), and the terrestrial snail *Paropea achatinaceum* (5.6%) (Table 1). Of flatworms collected many were of the *Bipalium* spp. (hammer-headed flatworms) and *Platydemous manokwari* (New Guinea flatworm), were also collected.

**Table 1:** The different species and numbers of each removed from each of the five different school locations are shown.

**School**	**Species name and numbers removed**												
	**ACH FUL**	**COR ASP**	**DER sp**.	**LAE ALT**	**LIM MAX**	**MEG sp**.	**PAL sp**.	**PAR MAR**	**PAR ACH**	**VER CUB**	**PLA MAN**	**BIP spp**.	**Snail**
Mt. View	0	0	1	1	2	2	11	0	213	237	1	3	0
Volcano	0	0	7	0	9	3	0	0	0	0	0	10	0
Kapoho	1	0	0	0	0	0	0	13	0	67	6	3	0
Laupahoehoe	0	0	0	30	1	0	0	15	19	36	4	15	0
Waimea	0	3,106	251	0	0	1	1	0	0	1	34	4	8
Total	**1**	**3,106**	**259**	**31**	**12**	**6**	**12**	**28**	**232**	**341**	**45**	**35**	**8**

*ACH FUL, Achatina fulica, giant African snail; COR ASP, Cornu apsersum, European garden snail; DER spp., Deroceras species including the gray garden slug and the marsh slug; LAE ALT, Laevicaulis alte leather leaf slug; LIM MAX, Limax maximus, leopard slug; MEG sp., Meghimatium sp., terrestrial slug; PAL sp., Pallifera sp., terrestrial slug; PAR MAR, Parmarion martensi, semi-slug; PAR ACH, Paropeas achatinaceum terrestrial snail; VER CUB, Veronicella cubensis, Cuban slug; PLA MAN, Platydemous manokwari, New Guinea flatworm; BIP spp., Bipalium spp, hammer-headed flatworm; Snail, unidentified small terrestrial snail*.

**Figure 3:**
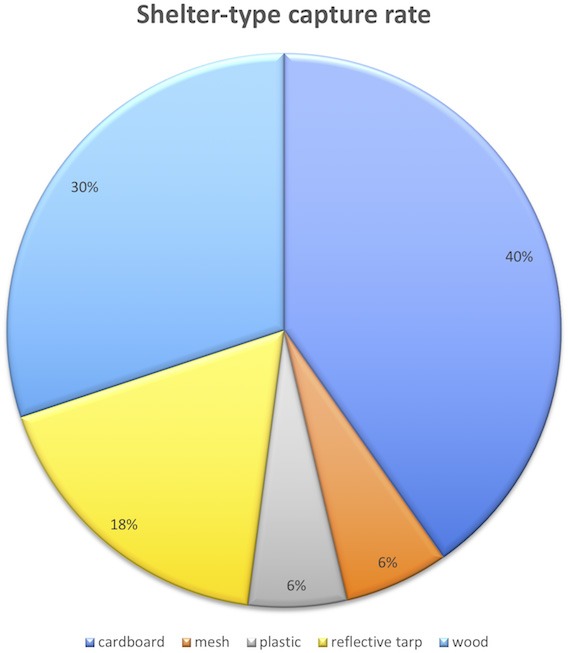
Recycled cardboard (40%) proved to be the most effective shelter followed by the raised wooden board (30%).

**Figure 4:**
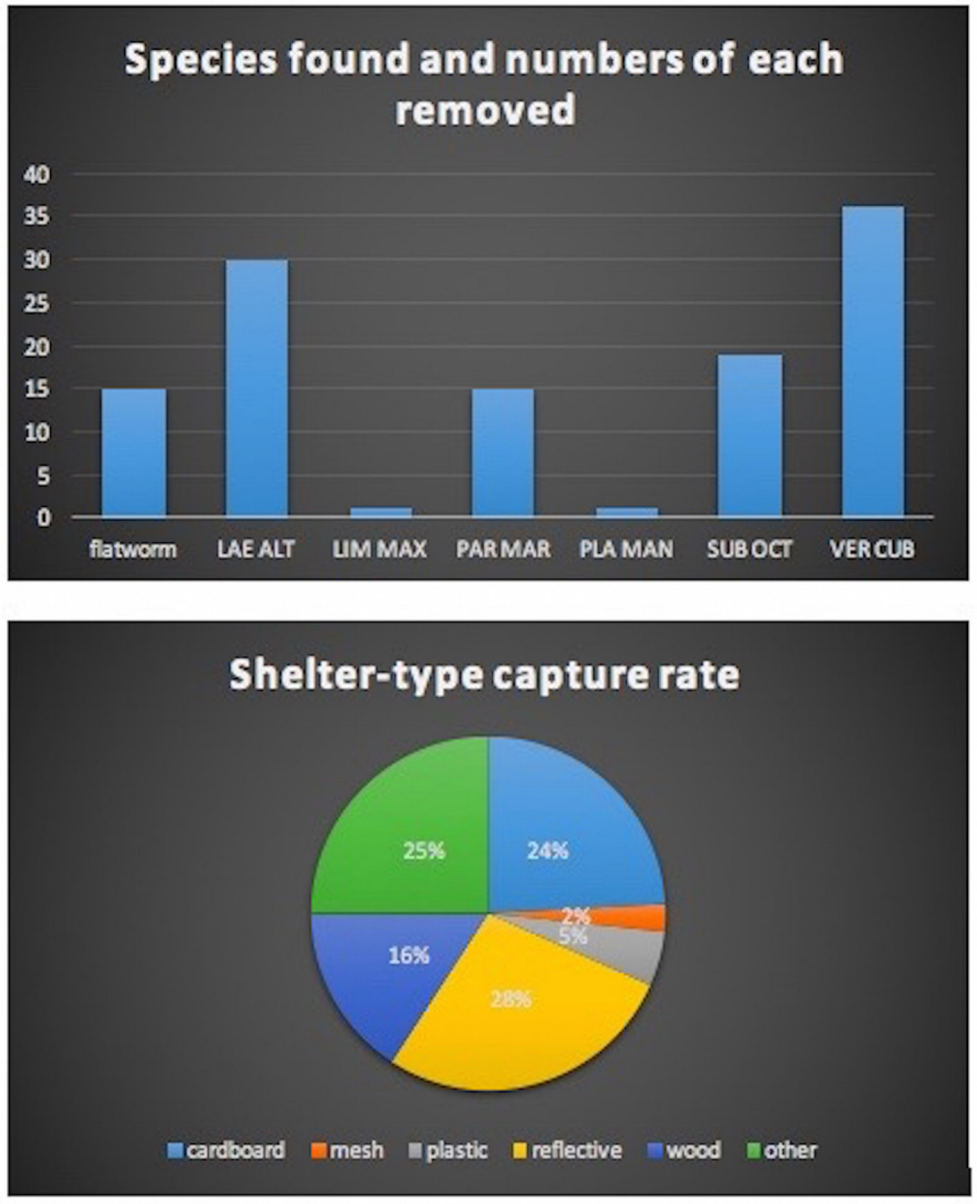
Use of bar graphs (above) to depict numbers of species found, and pie charts (below) to display shelter-type capture rate. This data came from a mid-elevation school.

Evaluation of shelter-type capture rate showed cardboard and a raised wooden board to be most effective (Figure 3). The greatest number of target organisms removed at one time from one shelter (wood) was 52. At one school the shelters were placed over wood chips that had been used between crop rows for mulch and this provided a desirable substrate that retained moisture and was highly attractive, especially to the Cuban slug. While the single layer of black plastic had a low capture rate a slug hunt revealed that a folded piece of heavy duty plastic or tarp made an attractive shelter for many slug and snail species and was especially attractive to the semi-slug. While the shelters were effective at harboring the target pests a large number were removed by hand collection from areas other than shelters, in part due to the infestation of the European garden snail at one school. At the low elevation school the substrate was primarily cinder and rough rock due to a relatively recent volcanic fissure eruption in 1960, and the substrate under the shelters was unattractive to slugs and snails. However slug and snail hunts were very successful in removing target organisms, students enjoyed the activity, and hunts are recommended as part of the management plan. Data collected was used to create pie charts of shelter-type capture rate, and bar graphs of species captured (Figure 4).

Three of the schools were located in the Puna District where many cases of angiostrongyliasis have originated. Multiple students from four of the schools knew someone, had a family member who had had angiostrongyliasis, or had a pet that had contracted the disease. An alumnus from one of the partner schools had been hospitalized with angiostrongyliasis the year before the project started. Multiple students at four of the five schools confirmed their families relied on rainwater catchment as their primary water source. Initially, teachers and students reported no or very limited knowledge of the rat lungworm parasite or the disease, but both groups showed great interest in the subject and became well-informed and eager to share what they learned. Classroom conversations revealed there is a great deal of misinformation in Hawai'i, such as washing produce with solutions such as vinegar to kill the rat lungworm parasite, which research shows is ineffective.

The use of the arts to convey information supported diverse student learning styles and abilities and encouraged the use of authentic assessments to evaluate student comprehension. The parasite life cycle lesson was taught using clay to model 3-D replicates of the host animals and the parasite. Reenactment of the life cycle using clay props aided student comprehension and promoted teamwork and communication. Students used video presentations to demonstrate how to properly prepare foods, especially headed vegetables to be eaten raw, and discussed cooking and freezing as methods for disease prevention. Classes from two schools created their own slug and snail shelters using found or recycled materials and based their designs on observation and critical thinking skills. Participating classes presented the information learned to non-participating grades with skits, puppet shows, posters, and informative talks. Students at one school designed and silk-screened T-shirts with a messages to control slugs, and made slug jugs with instructions for sale at a school event. A student at another school made an informational website on rat lungworm disease. Only one of the partner schools created an ArcGIS online account and students at this school worked in teams to produce a story map. The story mapping project supports language arts as each page must have content. A public forum with presentations by students and teachers from participating schools was held at UHH at the end of the school year, which was videotaped by a local public television station^3^ and written up in a local paper^4^ An ArcGIS story map on the project was published by the project leader^5^.

Rainfall data should be consistent, and we recommend that schools place a priority on the collection of weather data. The development of a more permanent wall poster of non-indigenous slugs and snails in Hawai'i is preferred over cards, which can be misplaced. Shelter maintenance and replacement could be supported as classroom projects, as can student experimention in the production and testing of shelters fabricated from other materials, including natural materials. The use of shelters for control expanded to other school garden projects. Students became adept at finding target species during gardening activities and hunts. Pests were removed and properly disposed of. Slug jugs were maintained and used at all participating school gardens. The practice has been adopted at other schools, and many students and teachers report using a slug jug at their residence. One gallon jugs have been replaced with five gallon buckets for schools with populations of large snails.

Teacher feedback was generally verbal and exchanged during school visits. Feedback received was consistently positive and in support of the program. Teachers stated the importance of the information learned and the opportunities it provided to expand student understanding of the scientific method, field work and data collection and analysis. They recognized the program as an effective method for community education. Students displayed great interest and developed their own student-led investigations. Students used the scientific method to test solutions for slug and snail control which included innovative ideas such as suspending potted plants in hanging containers and utilizing “hot” wires with electrical current supplied by a 9 volt battery. Teachers reported that their students shared the information learned through the project with their families. One teacher replied that she was called upon by family, friends, rat lungworm victims and friends of friends to answer the many questions and concerns they have about rat lungworm and the disease.

## Discussion

The project's overall success led to grant funding by the State of Hawai'i to create and write the IPM plan and rat lungworm disease-related lessons conceptualized during the pilot study and make the curriculum available online. Thirty additional classroom visits were made and seventeen teachers and classes participated in the materials trials. The IPM plan and lessons are now available online for all educators to download in PDF form. The lessons are aligned with Common Core Math, Next Generations Science, and Hawai'i Department of Education standards. There are 11 lessons incorporating STEAM subjects that are relative to the subjects of rat lungworm, invasive species, and disease prevention. A master data sheet and an evolving identification file are provided as supplemental materials^6^.

If this curriculum and management plan can be implemented at K-12 public schools throughout the state, students can assist in gathering data that can streamline control efforts and aid in prediction of possible angiostrongyliasis outbreaks. Collection of data by multiple schools in multiple locations on multiple islands over time will provide a valuable large data set, allowing for better understanding of intermediate host populations, mollusk infection rates, and effects of control efforts. Reporting using ArcGIS online would allow schools across the state to share information and experiences and to publish their results. This free technology is under-utilized in Hawai'i public schools. It introduces students to ArcGIS, a data and mapping software that is widely used in public planning and natural resource management and therefore is an excellent career track skill for students. There are educational videos to aid teachers and the technology is widely applicable for many subject areas. Schools should be encouraged to set up school-wide accounts.

A statewide project partnering with youth to advocate for public participation in host control may provide the strategy needed to reduce the very high infection rates we currently see in host animals. Additionally, school garden projects can serve as sentinels for early detection of newly arrived gastropod species, such as the semi-slug. Originally not seen at one of the mid-elevation schools, numbers of this species are now high, showing this important pest is now established in this location and may be capable of moving into higher elevation climates and ecosystems. This species has not yet been found on Kauai and every effort should be made to prevent its arrival. As students learn to recognize their regular local slug and snail populations they will be quick to identify a new arrival they are not familiar with.

With the increasing number of cases, the severity of the disease in Hawai'i, the widespread use of rainwater catchment, and the importance of consumption of fresh, local produce, this program fulfills an essential need. Integration of the methods developed here can add needed measures for school garden safety and promotion of community health. The dissemination of research-based information can help dispel misinformation that increases disease risk. The inclusion of epidemiology in education has been shown to be effective at improving the delivery of science and mathematics, increasing public health literacy, and providing a career pathway into public health (25). The CDC has a Science Ambassador Program, which provides training and support for middle and high school teachers for the development of lessons based on disease-related topics. In Hawai'i, integrating epidemiology and education could cover other diseases that are problematic, including other food borne diseases, common pathogens such as *Leptospira, Staphylococcus aureus* and methicillin-resistant staphylococcus, pathogens that can contaminate rainwater catchment systems such as Giardia, Salmonella and *Escherichia coli*, and other zoonotic diseases such as dengue, malaria, and zika.

This project serves as a template for other teacher and student-led investigations using place-based education to examine important community issues. Students displayed great interest in the diverse life-forms found under the shelters which included slugs, snails, flatworms, insects, spiders, millipedes, centipedes, and lizard eggs, and these types of experiences can spark a young persons interest in science. There is broad support for this educational project by school principals and teachers, parents, and the Hawai'i Farm to School Hui, which supports school garden networks on all of the islands. Because rat lungworm disease is a health issue, school garden projects are required to take steps to prevent disease. The materials for shelters are inexpensive or free, and the lessons are easily accessible for teachers. These factors make this project sustainable. However statewide implementation of the project will require funding for teacher training workshops and webinars, and student workbooks for grades 5–6 and 7–8 in the form of investigative journals. Assessments must be developed to track teacher, student and program success and all materials must be reviewed and approved by the Hawai'i Department of Education for this to become a statewide school program. The benefits of this program could be broad and far reaching.

## Conclusion

The educational materials developed through this project are globally unique and can be employed in other areas where *A. cantonensis* is found. The curriculum can be expanded for high school grades, which would provide for a career track in public health. As angiostrongyliasis is now understood to be a global emerging disease and the parasite range is expected to expand, this is a timely and worthwhile effort that can be replicated. There are school garden projects in the states such as Florida, Louisiana, and Texas where the parasite is found, and California could be proactive as the parasite is approaching from both east and west coasts. Engaging youth in public health can have long-term positive impacts in prevention of conditions that cause disease. We are growing a generation that understands rat lungworm in Hawai'i and the actions needed to prevent disease. The rat lungworm parasite and the threat of the disease in Hawai'i impacts agriculture, food security, tourism, and public health. Hawai'i must make every effort to properly address this problem to prevent the spread of the parasite to other shores.

## Author contributions

KH and SJ contributed to the conception, design, drafting, and revisions of this work. JB, MD, SF, and RH provided their classes and class time and assisted with curriculum modification, implementation, and review.

### Conflict of interest statement

The authors declare that the research was conducted in the absence of any commercial or financial relationships that could be construed as a potential conflict of interest.

## Footnotes

^1^http://files.hawaii.gov/dbedt/op/spb/Volume_II_History_of_Agriculture_in_Hawaii_and_Technical_Reference_Document_FINAL.pdf

^2^https://www.ed.gov/esea.

^3^http://naleo.tv/?s=Rat+LUngworm+Forum

^4^http://www.hawaiitribune-herald.com/2016/05/23/hawaii-news/keiki-get-jump-on-rat-lungworm-education/.

^5^https://uhh.maps.arcgis.com/apps/MapJournal/index.html?appid=5ccc2fa5cf7b441fa2711c5b60 e853b6&edit

^6^http://pharmacy.uhh.hawaii.edu/rlw/lessonplans.php
